# Cognitive Inference Device for Activity Supervision in the Elderly

**DOI:** 10.1155/2014/125618

**Published:** 2014-10-27

**Authors:** Nilamadhab Mishra, Chung-Chih Lin, Hsien-Tsung Chang

**Affiliations:** Department of Computer Science and Information Engineering, Chang Gung University, 259 Wen-Hwa 1st Road, Kwei-Shan Taoyuan 333, Taiwan

## Abstract

Human activity, life span, and quality of life are enhanced by innovations in science and technology. Aging individual needs to take advantage of these developments to lead a self-regulated life. However, maintaining a self-regulated life at old age involves a high degree of risk, and the elderly often fail at this goal. Thus, the objective of our study is to investigate the feasibility of implementing a cognitive inference device (CI-device) for effective activity supervision in the elderly. To frame the CI-device, we propose a device design framework along with an inference algorithm and implement the designs through an artificial neural model with different configurations, mapping the CI-device's functions to minimise the device's prediction error. An analysis and discussion are then provided to validate the feasibility of CI-device implementation for activity supervision in the elderly.

## 1. Introduction

A cognitive inference device (CI-device) may be defined as a smart portable device embedded with smart cognitive programs that mimic human perceptions, judgments, thinking, and other cognitive functions to enhance the inference capabilities of the device. Human intelligence is natural and sufficiently standard to accomplish self-regulated physical and mental activities; however, increasing chronological and physiological age may degrade these physical and mental utilities, which ultimately leads to the decay of physical functions and natural intelligence in the course of aging. Activity supervision in the elderly is a tedious task for any young individual; a CI-device may instead be employed to manage this task. Activity supervision encompasses activity administration, management, assistance, and control, which become much more essential for older individuals to lead a self-regulated life in their own home; that is, instead of self-reliance, the elderly must rely on the CI-device to continue day-to-day activities smoothly. In the CI-device, human intelligence can be mapped onto a machine intelligence to act as a smart assistive device for the elderly. Human activity and life span are enhanced by advances in science and technology. The elderly population is concerned about the progress of the socioeconomic environment and advancements in research in medicine and health, which could prolong the normal human life span and improve the quality of life. In major, economically established countries, the percentage of the population that is elderly is expected to rise to 25% of the total populations by 2030 [1]. Thus, specific wellness applications need to be developed to determine the status of the elderly individual's daily activities in terms of leading a self-regulated life [2, 3]. In addition, activity-tracking systems may be developed to track and gently notify the elderly of deviations in activity without compromising privacy [4].

**Figure 1:**
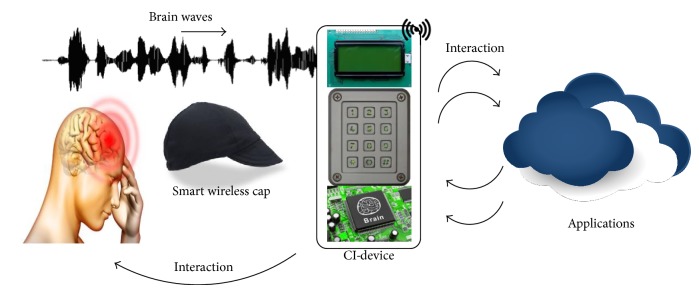
CI-device interacting with an elderly individual and external applications.

Different types of brain wave patterns may be identified in an elderly brain in accordance with elderly activities: alpha, beta, gamma, theta, and delta patterns [5]. In the context of using a CI-device, elderly individuals use a wireless electroencephalography (EEG) data acquisition system on a smart cap to record their brainwaves directly through their hair and transmit them to the CI-device for further analysis and exploration. The smart cap uses wireless micro-EEG sensors to provide adequate autonomy and comfort to the elderly without the threat of critical data loss. The CI-device can accept wave patterns from the elderly brain, analyse those patterns to identify thoughts and activities, and interact with the elderly and external applications as described in Figure 1. Thus, the CI-device acts as an assistive interface between the elderly individual and the environment and external world. The identified activities of the elderly can be classified into different activity sets based on their characteristics and behaviours. The activities of the elderly can be broadly classified into three types: periodic activities in the elderly, aperiodic activities in the elderly, and sporadic activities in the elderly. Periodic activities are normal daily activities that are performed at regular intervals, such as a morning walk, bedtime bath, breakfast, lunch, dinner, and sleeping at night. Sporadic activities may be defined as irregular periodic activities, for example, taking medicine during the week, and aperiodic activities are unstructured and nonperiodic activities, for example, reading books or watching movies. In general, a periodic activity can be treated as a common activity; however, the sporadic and aperiodic activities are signified as exclusive elderly activities due to individual variations.

In reality, a CI-device cannot think as humans think, but it can give expert opinions to the elderly based on intelligent information previously embedded in the device. The current research trend encourages researchers to transform human intelligence into device intelligence so that the device can act as if it were human. Researchers can use intelligent tools to design and train the artificial neural system on the CI-device to incorporate human perception, thinking, and judgment in such a way that no gap can be distinguished between natural (human) and computational (machine/device) intelligence. In this work, we discuss a smart device design framework accompanied by an inference algorithm to embed human intelligence into a CI-device for self-regulated activity supervision in the elderly. In the design framework, we mainly discuss the functional analysis and modelling of the CI-device followed by a device learning process through a fuzzy-based backpropagation neural network algorithm (FBN-algorithm). We also conduct a broad review to identify systems or devices and their functions or operations that have already been examined or developed for activity supervision in the elderly.

The overall organisation of this paper is as follows.  Section 2 discusses the related work in activity supervision in the elderly with the assistance of the latest tools and devices. Our proposed CI-device design framework along with its inference algorithm is discussed in Section 3.  Section 4 highlights the analysis and discussion of the proposed CI-device aimed at wide implementation in activity supervision in the elderly. Finally, Section 5 presents our conclusions.

## 2. Related Work

Though a substantial amount of healthy aging research has been completed or remains in progress, there is still a lack of appropriate cost-effective cognitive solutions to effectively solve the real time hazards of aging in society. The progressive growth of the elderly population worldwide will create serious consequences in the near future. Day by day, with the advancement of science and technology, healthcare systems are being developed to allow the elderly to maintain self-regulated lives in their own homes. A smart power-monitoring device has been proposed that assists the elderly to identify and regulate home electrical appliances used for daily activities [6]. Basically, in activity supervision systems in the elderly, sensors and actuators have widespread and ubiquitous applications in real-time home monitoring systems to care for the elderly [7]. Advances in sensor technology enhanced with advanced health care systems for the elderly provide home medical assistance to disabled elderly to allow them to perform their self-regulated activities securely at home [8]. The main goal of smart home monitoring systems for the elderly is to provide a highly cost-effective, safe, and secure solution for the purpose of wellness [9]. The work in [10] has focused on the development of an electronic monitoring device to detect illness in the elderly and alert the individual at the receiving end to immediately perform the necessary remedial actions. An infrared (IR) sensor-based activity-monitoring device for the elderly is proposed in [11] to detect and monitor abnormal elderly activities and behaviours in a home-based healthcare environment. The work in [12] proposes a simple health care-monitoring system for the elderly by considering the design of both the software and hardware components that can be successfully applied in health community services for the elderly. The use of visual sensors in both in-home and community-based activity monitoring systems may compromise privacy and security; however, it is realistically simpler and easier to embed them into the elderly individual's living environment. The work in [13] concentrates on the use of visual sensors in an older individual's living environment to allow care professionals to efficiently observe the individual's activities and to take immediate remedial actions in case of any serious activity disorders. With the evolution of the internet of things (IoT), each and every device will soon have communications abilities. Thus, the authors in [14] propose the use of the IoT in a smart home-monitoring framework for remote monitoring and control of household appliances, so that the elderly can easily lead an independent life. Life is tedious for elderly individuals who suffer from dementia, so a system is proposed in [15] to monitor the behavioural situation of such elderly patients in terms of position localisation and motion. Cognitive science studies human natural intelligence, that is, human perception, conception, and judgment capability. Such intelligence can be mapped to a cognitive sensor network to develop a smart home-monitoring system for the elderly. Such an intelligent system can perform various complex functions, such as detecting abnormal behaviour and remotely monitoring home appliances, that is, monitoring electrical appliances, water use, and the bed [16]. The work in [17] recommends a generic approach to develop intelligent ambient devices for managing agile and complex human behaviours for application in a wide variety of domains.

However, very few prior studies address any use of cognitive intelligence devices for an activity and behavioural supervision system that is able to assist the elderly in maintaining a self-regulated life. Thus, we introduce a CI-device for activity supervision in the elderly to address the gap between natural intelligence and device intelligence. To our knowledge, this is a new alternative effort in the direction of activity supervision in the elderly using a CI-device.

## 3. CI-Device Design Framework

To design a CI-device for activity supervision in the elderly, we present a pattern-inference cycle for pattern recognition in the elderly, an activity-inference framework for activity identification in the elderly, and an FBN-algorithm for device learning to ensure functional and operational efficiency. We broadly classify this section in three stages. Stage-1 highlights the general functional analysis of the CI-device. Stage-2 includes the functional modelling aspects needed to construct an intelligent device design framework. Stage-3 describes the learning process of the CI-device. The learning process ensures that the CI-device will obtain the necessary knowledge to supervise the elderly in almost all expected circumstances.

### 3.1. Stage-1 (Functional Analysis)

**Figure 2:**
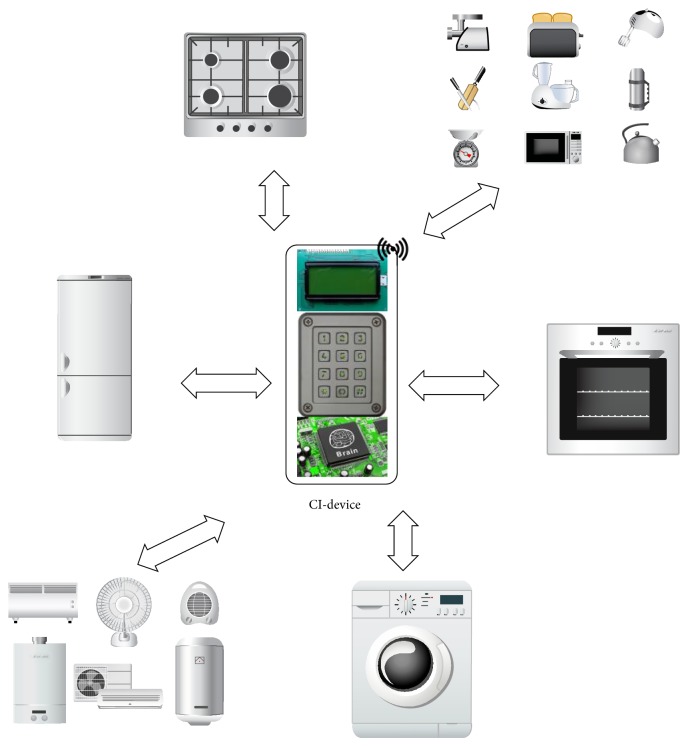
CI-device remotely monitors different household equipment attached with smart sensors.

The functional analysis stage of the CI-device aims to explore the feasibility of the expectations of the elderly. The CI-device may be employed as a cluster controller in a smart home-monitoring cluster in addition to providing activity supervision of the elderly. Therefore, the device remotely monitors different sensors attached to household equipment, such as the gas metre, pressure sensor, electrical metre, and smoke detector. The CI-device extracts data from these sensors, transforms the data into tactical knowledge, and informs the elderly about their operational status (see Figure 2). The CI-device also acts as a decision maker to regulate the sensors attached to the household equipment [28] and will be a useful tool to allow the elderly to remotely monitor household equipment without any physical inspection. The Zig Bee module can be fabricated in a CI-device to interact with those sensors [29]. The CI-device should have a smartphone-like display to visualise the status of individual household equipment and a USB interface to connect to external peripherals. The EEG-regulated smart home-monitoring system can supervise the smart sensors attached to the household equipment. For an EEG-regulated smart home-monitoring system, the EEG data-intensive CI-device may be designed to interpret and decode the older individual's thoughts and intentions to generate the instructions to control the sensors attached to the household equipment. The IoT is an evolution of wireless access and network technology [30]. With the intervention of IoT technology in a wireless smart home-monitoring system, the IoT chip in every household appliance can be designed to transform it into an IoT object. The IoT object may have the ability to co-ordinate with the CI-device when required to transmit its status and other information. Thus, the interaction between household IoT appliances and CI-device allows the elderly to more easily perform their activities of daily living. Overall, the CI-device should support the following functions to assist the elderly.

### 3.2. Functional Expectations for a CI-Device in the Elderly

- It tracks activity in the elderly before and after completion.
- It gently reminds the individual of any activity if it is not completed in time or any deviation from the normal standard is identified.
- It accepts the individual's brain wave patterns and assists in some thinking, memorising, and memory-recall processes.
- It supports the elderly individual's decision-making process.
- It coordinates all of the elderly individual's activities effectively.
- It links with external applications for effective cooperation and interactions.
- It coordinates all of the sensors attached to household equipment, informs the elderly individual in cases of need or emergency, and performs any necessary actions.
- In case of sickness, the CI-device should generate phone calls and messages to the healthcare centre or well-wishers of the elderly individual to take instant counteractive actions.

Due to its intelligence and self-learning capabilities, the CI-device should carefully monitor the elderly individual's activity and make suggestions when required. For example, if the elderly individual forgets to switch off the heater after cooking, then the CI-device either should gently prompt the individual to switch off the heater or should direct the electrical sensor attached to the heater to disconnect. To determine the probability of device usage in the elderly based on their ability to perform daily life activities, real-time observation data have been collected. Let C1, C2, and C3 be three clusters of elderly individuals such that C1 includes those individuals who perform all of their daily life activities without any device assistance, C2 includes those individuals who perform all of their daily life activities with standard device assistance, and C3 includes the individuals who perform their daily life activities with major device assistance. We find the migration rate of the elderly from one cluster to another cluster by computing the transition probability of present-period elderly individual's interest with respect to the next-period elderly individual's interest as described in Table 1.

Table 1 indicates the consistency of an elderly individual's interest within consecutive periods, with a trend toward an increase in the probability of interest in device usage as the individual ages, as the individual begins to rely on the support of a CI-device to conduct their daily life activities.

### 3.3. Stage-2 (Functional Modelling)

**Figure 3:**
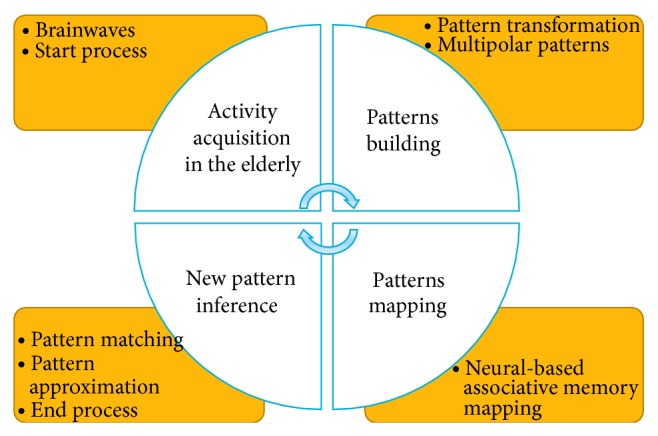
Pattern-inference cycle.

Here, we discuss pattern-inference cycles for the purpose of activity recognition in the elderly. A pattern-inference cycle is the process of extracting new patterns from a storehouse of associated patterns that are encoded in certain forms. When the storehouse is triggered by a pattern, the associated pattern pair is mapped, which can be accomplished by associating the previously stored patterns with currently generated patterns to infer the new pattern. The pattern-inference cycle (see Figure 3) for the proposed CI-device assists elderly who are suffering from amnesia by inferring new patterns. The pattern-inference cycle begins by receiving the brainwave activity of the elderly, builds the corresponding activity patterns, maps the pattern to the stored patterns, and further infers new patterns based on approximation and partial matching. A neural-based associative memory mapping mechanism may be embedded in the CI-device to execute the pattern-inference cycle; however, the self-directed functioning of the CI-device based on the behaviours of the individual is an important cognitive measure to actually identify the in-time requirements for ambient device assistance in the elderly.

Some text or image patterns related to the activities and behaviours of the elderly may also be stored in the CI-device, and by viewing the stored patterns, the elderly individual may be able to recognise the new patterns. The CI-device recommends new approximate patterns to the elderly through its inference capabilities to meet the individual's requirements in terms of thinking, memorising, and memory recall. Mostly, the pattern inference in the CI-device supports the elderly in recalling their historical activities and reminding them of current activities based on partial research or matching. The activity-inference framework may use the pattern-inference cycle to analyse historical activity data to infer new activity patterns in the elderly individual's behaviour.

### 3.4. Purpose of Using the Activity-Inference Framework

- To identify the activities and behaviours of the elderly individual through brainwave patterns.
- To explain the interactions and relationships between the elderly individual and the CI-device.
- To design a controller based on the activity of the elderly individual and the device's functional and operational capability.

### 3.5. How the Activity-Inference Framework Functions

The activity-inference framework functions in two steps. Here, our aim is to design a mathematical model of activity in the elderly and estimate the activity fitness and ranking.

*Operational Function*. The activity-inference framework determines a set of operational activity models for the target CI-device from which the most suitable model can be obtained. The activity model in the elderly is denoted by an operational function, *y* = *f*(*A*, *θ*), where *y* = the output of the activity model, *A* = the input activity vector, and *θ* = the corresponding fitness value.

*Fitness Selection*. Once the structure of the activity model is known, we can apply a genetic algorithm-based optimisation technique to determine the fitness vector *θ*. The fitness selection is performed through choosing the *θ* that best fits the activity dataset. For instance, let *y*_1_ = *f*(*A*_1_, 5) in a 10-point fitness scale for an elderly individual, which shows that the elderly individual is 50% fit to perform activity *A*_1_; that is, the activity precision = 0.5.

### 3.6. How to Rank Activities in the Elderly

The activities in the elderly can be ranked based on fitness value. Let *X* be a fitness function and *A* be an activity in the elderly such that *X*(*A*) = *a* fitness value. Given four activities *A*_1_, *A*_2_, *A*_3_, and *A*_4_, they can be ranked based on their fitness values such that *X*(*A*_1_) = 5, *X*(*A*_2_) = 3, *X*(*A*_3_) = 7, and *X*(*A*_4_) = 6 as described in Table 2.

The number of activities depends on the elderly individual, and the activity selection can be made based on ranking; however, two activities with the same fitness value may have the same ranking. The CI-device uses this system to assist the elderly individual by predicting and reminding the individual of new activities. Additionally, in some cases, the CI-device will be able to predict at what time the elderly individual will perform each activity [31]. A three-step process can be used in the activity-inference framework.

*Step 1*. Record the activities of the elderly individual through brainwave patterns.

*Step 2*. Analyse those activities using an inference algorithm.

*Step 3*. Predict new activities and gently remind the elderly individual in the case of minor or major activity deviations.

The system is suitable for forecasting the periodic and sporadic activities of the elderly individual through the CI-device; however, it will be highly complicated for aperiodic activities.

*Inference Algorithm* 1. The activity-inference algorithm (Algorithm 1) is a self-regulated intelligent algorithm that can be embedded into the CI-device to create automatic alerts for the elderly individual. While designing the inference algorithm, we must ensure that a dead brain cannot generate any wave patterns. *P*_1_, *P*_2_, *P*_3_, *P*_4_, and *P*_5_ are the alpha, beta, gamma, theta, and delta brain wave patterns, respectively, that act as stored patterns for the CI-device to perceive further patterns for analysis and exploration. Algorithm 1 shows how the CI-device can automatically recognise the elderly individual's activity based on inferred brainwave patterns and shows how it can check the elderly individual's needs for assistance. Step 2 of Algorithm 1 mainly performs functional processing, such as pattern transformation, pattern mapping, activity fitness estimation, emergency determination, and other relevant tasks. The CI-device, after receiving the brainwaves, transforms them into standard bi-polar patterns to perform effective mapping between the current brainwave patterns and stored patterns to infer new approximate patterns for the elderly individual's ongoing activities. The CI-device does not hamper the privacy of the elderly individual while undisturbed; however, during sleep, the CI-device can also perform EEG monitoring to determine the overall safety of the elderly individual. In a real-world implementation of activity supervision in the elderly, an elderly individual can comfortably use a smart cap that consists of a wireless EEG monitoring system such that the sensors can cooperatively record brainwaves directly through the hair and send the brainwaves wirelessly to the CI-device to establish the interactions and relationships between the elderly individual and the CI-device [32]. The wireless EEG-acquisition system and mobile EEG data-recording system have a significant role in recording and transmitting the brainwaves from the elderly individual to the CI-device without compromising the expected privacy of the elderly individual [33, 34]. We can incorporate the CI-device's functions into a smartphone/watch-like portable device for convenience. In Algorithm 1, the relative values of *T* and *θ* can be measured through the operational function and fitness selection of the activity-inference framework. We consider five broad activity categories in the elderly to cover the range of activities of daily living. These activities are judgment (active thought activity), work (active physical activity), undisturbed (active ideal activity), pensive (active stress activity), and sound asleep (passive physical activity). All periodic, aperiodic, and sporadic elderly activities also come under this five-activity classification strategy. The SVM (support vector machine) based supervised learning mechanism may also provide a decent solution for the effective classification of elderly activities based on certain behaviours and characteristics [35]. The efficiency of the CI-device is heavily dependent on the precision of the activity-inference system; that is, a higher inference accuracy improves the device's efficiency. Based on the above discussion, we see that three influential parameters can be used to assess the CI-device efficiency: activity accuracy, activity fitness (*θ*), and inference accuracy.

### 3.7. Stage-3 (Learning)

In this stage, we mainly discuss the learning mechanism of the CI-device to make it suitable for activity supervision in the elderly.

### 3.8. FBN-Algorithm

Here, we focus on the device's learning system using the FBN-algorithm. The neural network in the CI-device can be trained through the FBN-algorithm to perform various complex functions, such as pattern recognition, system control, and activity identification and classification, and overall, it acts as an artificial brain within the CI-device. The FBN-algorithm provides the knowledge-acquisition system of the neural network embedded in the device through the following steps.

*Step 1*. Design input/output (*I*/*O*) data sets by considering historical activities in the elderly individual.

*Step 2*. Configure the neural network architecture (NN0, NN1, and NN2).

*Step 3*. Configure the type-1 fuzzy weight matrix for the input-to-hidden and hidden-to-output layer in the range [−1, + 1].

*Step 4*. Train the neural network by tracing 60% of the *I*/*O* data sets until the error is acceptably low. During training, type-1 fuzzy weights are initially assigned, and supervisory weight adjustments are then performed to minimise the functional learning error of the CI-device.

*Step 5*. Test the neural network by tracing 40% of the *I*/*O* datasets to minimise the functional testing error of the CI-device.

*Step 6*. Once the test process is acceptable, use the neural network in actual CI-device implementation to perform unknown complex operations. The device's learning ensures the self-learning capability to build intelligence in the CI-device for self-regulated activity supervision in the elderly.

## 4. Analysis and Discussion

Here, we discuss some empirical suggestions to analyse the proposed CI-device for the purpose of wide implementation. If we can develop a small portable device embedded with an intelligent design framework, then it will be sufficiently convenient for the elderly to introduce it into their daily living environment. The CI-device mainly emphasises the use of an intelligent framework, that is, a pattern-inference cycle and activity-inference framework followed by an FBN-algorithm for the smooth supervision of activity in the elderly. Our work analyses the effort to design a CI-device that assists elderly individuals with maintaining their activities of daily living through augmenting and supplementing their cognitive functions.

We broadly divide the analysis and discussion context into two major phases. In phase-1, we perform some activity-level analyses by considering the daily living activities of the elderly, and in phase-2, we emphasise the implementation analysis of the CI-device in the daily living activities of the elderly. However, prior to the analyses, some previous works are reviewed to ensure the importance of the system and device investigations to the associated functions in the direction of activity supervision in the elderly. A detailed review is presented in Table 3. The review's details clearly indicate the keen interest of researchers in either developing a system or designing a device for the purpose of assisting the elderly.

Device cognition is a challenging issue for current researchers, given the rapid advancement of medical engineering and instrumentation. In our work, we suggest using a cognitive device design framework in a CI-device to enable human-like inference capability for use by the aged. Due to its intelligence, the device may also cooperate with the social privacy systems of elderly individuals.

### 4.1. Phase-1 (Activity-Level Analysis in the Elderly)

**Figure 4:**
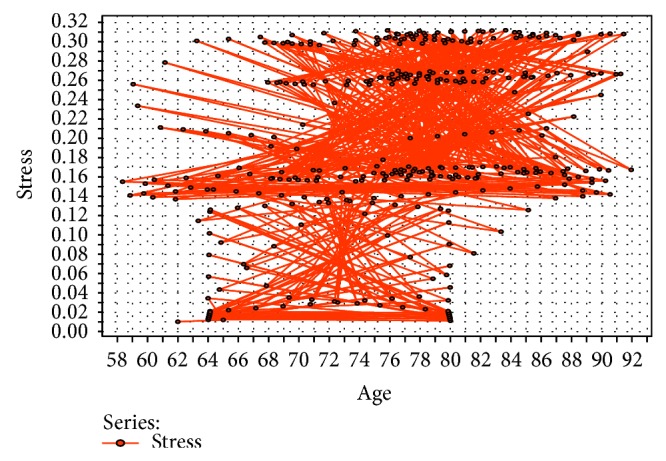
Stress-level analysis in the elderly based on age clusters.

**Figure 5:**
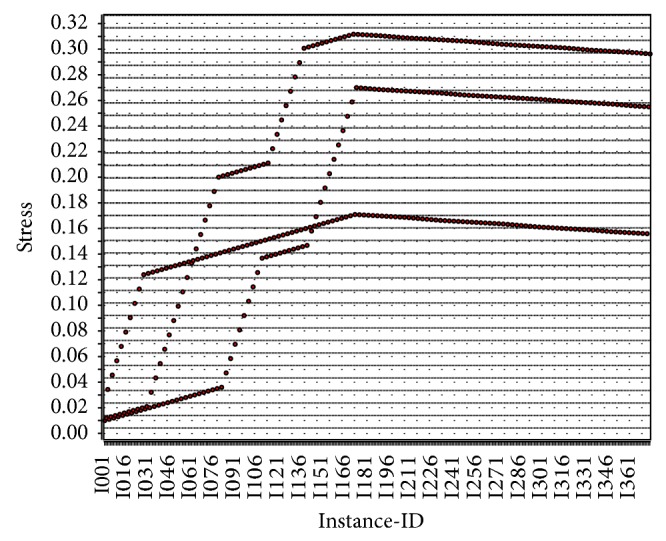
Stress-level analysis in the elderly with respect to the number of instances.

**Figure 6:**
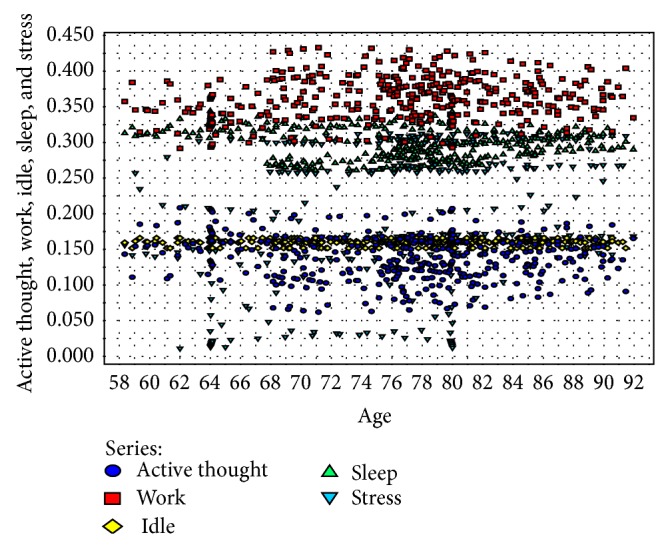
Complex inference patterns of activity-level analysis in the elderly.

Here, we want to visualise exactly how patterns are generated from data sets on the activities of the elderly. Some activity-level analyses in the elderly are conducted by considering five broad activities of the elderly to incorporate all of the common activities of daily living. These broad activities are active thought, work, ideal, sleep, and stress. Stress in the elderly is a psychologically pensive activity, and resting stress levels are an important measure to determine the wellness of the elderly in an effective way. In Figure 4, we perform a stress-level analysis based on clusters of the elderly and observe that individuals in the age cluster of 67–87 experience more stress, which leads to various diseases. Thus, the CI-device, through brainwave analysis, should identify mental stress in the elderly to reduce potential risks. In Figure 5, we perform a stress-level analysis in the elderly with respect to the number of instances and find increased mental stress in a series of instances. In Figure 6, the complex inference patterns of activity-level analysis in the elderly are grouped, building on those associated activities. The patterns are analysed in the context of the activities of the elderly, and statistical inference is used to identify abnormalities and outliers in the activity patterns. In Figure 6, the tiny coloured spaces define the activity patterns and the corresponding parameters of the associated activities. The real-time data are collected by employing smart sensing objects and provided to a data miner to obtain statistical inferences, as described in Table 4. The real-time data for the elderly can be normalised and scaled into a fuzzy-associated data system using the standard data range [0, 1]. To analyse the activity level in the elderly, their mobility factor must be considered. Thus, to scale the results to a mobility factor, we consider three quantifiers, that is, 8: high mobility, 6: average mobility, and 4: lowest mobility. Deviation is a common factor due to irregular activities in the elderly. Thus, by analysing Table 4, predictive inferences can be made regarding the wellness of the elderly. The inference logic indicates that no greater deviation occurs if the daily life activities of the elderly are periodic with respect to the number of instances, which is unrealistic for the elderly individual because in reality, the activities combine periodic, aperiodic, and sporadic activities.

### 4.2. Phase-2 (CI-Device Study Analysis)

### 4.3. Statistical Study Analysis

Based on the activity inference algorithm in the elderly (Algorithm 1), we consider five elderly functional activities (*A*_1_, *A*_2_, *A*_3_, *A*_4_, and *A*_5_) for study: *A*_1_← judgement (active mental activity), *A*_2_← work (active physical activity), *A*_3_← undisturbed (active ideal), *A*_4_← stress (active pensive), and *A*_5_← sound asleep (passive ideal). In addition, to measure the performance of the CI-device, four performance measurement parameters (*P*_1_, *P*_2_, *P*_3_, and *P*_4_) are considered as follows: *P*_1_← activity precision, *P*_2_← activity fitness, *P*_3_← inference accuracy, and *P*_4_← functional efficiency. Based on the functional activities and performance measurement parameters, a real-time empirical study is conducted in the elderly, considering the device's functions to obtain statistically correlated data sets. The final statistical report is described in Table 5. Here, we consider the elderly individual's natural cognitive discrimination level (CDL) (good: 8, average: 6, and poor: 4) to compute the definite statistical assessment instances. Based on statistical analyses of Table 6, we can ensure approximately 89.880% functional efficiency for our proposed CI-device; however, this value can be improved by improving the other parameters. Here, we compute the device efficiency for each activity by taking the accuracy average of *P*_1_, *P*_2_, and *P*_3_, and the final average accuracy for all of the activities can be computed to estimate the approximate efficiency of the CI-device.

The average functional efficiency of the CI-device is directly proportional to the CDL value (see Table 6). Thus, based on the CDL, the physical ability level, and the mobility level of the elderly individual, the CI-device may be introduced either as a smartphone/watch-like portable device or as a smart cognitive robotic device to regulate the elderly individual's activities and provide the necessary assistance without any human interventions.

### 4.4. Computational Study Analysis

**Figure 7:**
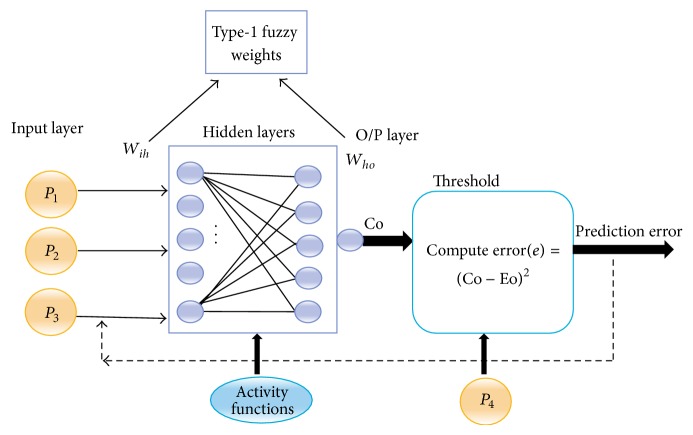
An NN2 (3-5-5-1) architectural model.

This phase discusses the computational analysis of the CI-device by mapping the possible device functions onto the neural network platform to minimise the device's prediction error. Once the CI-device is embedded in the smart inference framework, an appropriate device learning process should be initiated to test the accuracy level. We apply a type-1 FBN-algorithm, in which the type-1 fuzzy weight matrix (*W*) can be estimated using a standard formula; that is, *W* = ∑(*P*_*i*_ *W*_*i*_)/∑(*P*_*i*_), where *i* = 1, 2,… [36, 37]. The FBN-algorithm is a type of supervised learning algorithm that inherits the characteristics of an artificial neural system. We map the activity data sets in the elderly onto the FBN-algorithm to obtain the device prediction error result using MATLAB neurofuzzy system environment. The fuzzy system environment has much more real-time control to regulate numerous sensitive applications [38]. So, we use a neurofuzzy environment to simulate the different neural network configurations through the fuzzified data sets relating to the elderly activities of daily living. In Table 7, we do not consider the link errors but instead focus on computing the prediction errors based on the computed and expected outputs correlating with the functional efficiency of the CI-device. In the statistical study analysis, a particular data set in the elderly is normalised for this problem, and we use this FBN-algorithm to compute the input, hidden and output neuron computations along with the prediction error for each step. To implement the FBN-algorithm, three neural network models are used, that is, NN0: a neural network with zero hidden layers, NN1: a neural network with one hidden layer, and NN2: a neural network with two hidden layers, as shown in Figure 7. A type-1 fuzzy weight matrix is used based on the inference algorithm for the inputs of each neuron. The inference algorithm can be used as a knowledge base to provide facts from the neurons in the form of linguistic control rules [39]. Let *W*_*ih*_ be the type-1 weight matrix from the input layer to the hidden layer and *W*_*ho*_ be the type-1 weight matrix from the hidden to the output layer. We consider the *P*_4_ parameter values of Table 5 as the threshold for the expected output (Eo), and the Co is the computed output from the neural network.

To determine the functions of the hidden layer, the five activity functions in the elderly are mapped to this layer. We use a sigmoid function as the transfer function to the network model, that is, sigmoid function (Φ(*I*)) = (1/(1 + *e* ^−*λI*^)), because the sigmoid function is a very common function compatible with the type-1 fuzzy-based neural network systems. Eta(*η*) is the learning rate, in which two values are considered, that is, 0.1 and 0.5, the momentum co-efficient (*α*) = 0, and the sigmoid gain (*λ*) = 1 for this problem. Because of difficulties in implementing a type-2 fuzzy data set associated with activity in the elderly, we implement a type-1 fuzzy data set associated with activity in the elderly to compute the fuzzy weight matrix. In this analysis, the different configurations of artificial neural networks (NN0, NN1, and NN2) are mainly designed and traced to the elderly activity supervision application to find out the relative performance through error prediction during functional training and testing of CI-device [40].

A neural network without a hidden layer (NN0) may not be suitable for complex problem computations. Based on the analysis of the results, the success of neural network architecture depends heavily on the availability of an effective learning algorithm. The speculative strength of the FBN-algorithm can be used in other applications to compute error-prediction results. The use of a single data set makes it difficult for the model to recognise trends and patterns that exist in the data. The implementation of the NN2 model in the CI-device yields the lowest average prediction error, that is, lowest AP-error = 0.02278, compared with the other models, and increasing the learning rate may improve the device accuracy. In addition, the analysis indicates that the AP-error can be minimised by increasing the number of functions in hidden layers. The type-2 fuzzy weight updating mechanism may minimise the prediction error of the CI-device by dealing with more uncertainties to increase the desired functional efficiency.

## 5. Conclusions

In this paper, we propose an intelligent device design framework that can be implemented in a CI-device to manage activities in the elderly in an effective and efficient way. We also design an inference algorithm that can be embedded into the CI-device to build its intelligence. Due to intelligence and self-learning capabilities, the device can cooperate with its social environment without hampering social security and privacy. Furthermore, the device may be a good companion for the elderly to help them lead a self-regulated life. In our work, we have added an absolutely new case in point for the aging activity level analysis, in which the activity data sets are normalized and transformed into standard fuzzified data sets having more than one thousand activity instances in the range of [0, 1]. The self-directed functioning of CI-device based on the aging behaviours is an important cognitive measure to actually identify the in-time aging requirements for ambient device assistance. Here we mimic the details of cognitive functional measures of CI-device that acts as middleware oriented cognitive interface in between the natural intelligence and device intelligence. As the FBN-algorithm inherits the topographies of computational intelligence, so we train and test the functional measures of CI-device through this algorithm with an aim to reduce the AP-error so as to achieve the desired efficiency. In future work, we will study firmware-updating mechanisms for the CI-device to improve its functionalities, power efficiency, and reliability as well as safety measures for activity supervision in the elderly. We also aim to implement a type-2 fuzzy weight updating mechanism to improve the functional efficiency of the CI-device through effectively minimising the device prediction error.

## Figures and Tables

**Algorithm 1:**
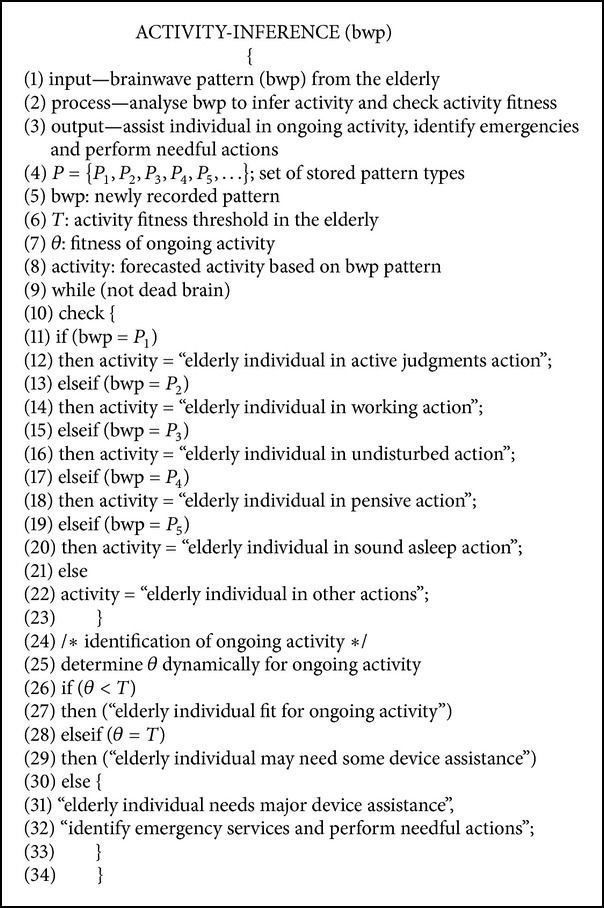
Algorithm for activity-inference from brainwave patterns in the elderly.

**Table 1:** Elderly individual's interest transition probability based on statistical observation.

Present-period ↓	Next-period →		
	C1	C2	C3
C1	0.25	0.66	0.09
C2	0.15	0.75	0.10
C3	0.09	0.16	0.75

**Table 2:** Activity ranking in the elderly.

Fitness value	Activity model	Rank
*X*(*A*_1_) = 5	*y*_1_ = *f*(*A*_1_, 5)	*R*(*A*_1_) = 3
*X*(*A*_2_) = 3	*y*_2_ = *f*(*A*_2_, 3)	*R*(*A*_1_) = 4
*X*(*A*_3_) = 7	*y*_2_ = *f*(*A*_3_, 7)	*R*(*A*_1_) = 1
*X*(*A*_4_) = 6	*y*_2_ = *f*(*A*_2_, 6)	*R*(*A*_1_) = 2

**Table 3:** Comparison of various works that propose systems/devices for activity supervision in the elderly.

Name of works/authors	Proposed system/device	Function/assistance
Kelly et al. [14]	IoT device	assists elderly to regulate household appliances

Gill et al. [6]	smart power-monitoring device	assists elderly to regulate home electrical appliances

Gaddam et al. [16]	cognitive sensor device	monitors home appliances for the elderly

Malhi et al. [10]	electronic monitoring device	detects illness in the elderly and alerts others

Zhou et al. [13]	visual sensor device	observes activity in the elderly to take immediate actions

Shin et al. [11]	IR motion-sensor device	detects abnormal activity in the elderly

Bosse et al. [17]	ambient intelligent device	fall detection in the elderly

Hervás et al. [18]	assistive navigation system	activity monitoring in the elderly and potential situation detection

Costa et al. [19]	ambient assistive system	creates an ecosystem of service and devices for the elderly

Ye et al. [20]	fall-detection device	detects activity acceleration to detect falls in the elderly

Andreoni et al. [21]	wearable sensor device	online activity monitoring and fall detection for the elderly

Jia et al. [22]	chair-based apparatus connected to a mobile apps system	health monitoring in the elderly

Gokalp and Clarke [23]	telemonitoring system	monitors activity and health in the elderly

Krishnan and Pugazhenthi [24]	assistive robotic device	enables self-transfer lifts in elderly patients

Chernbumroong et al. [25]	assisted living system with multisensor devices	activity monitoring in the elderly

Botia et al. [26]	ambient assisted living system	detects abnormal situations in the elderly

Costa et al. [27]	visual E-care system	(i) prescribes physical exercise for the elderly (ii) monitors physical activity and health status in the elderly

Our work	CI-device	(i) accepts brainwave patterns for activity monitoring in the elderly (ii) human-like inference capability (iii) uses activity-inference algorithm with FBN-algorithm to allow device cognition (iv) acts as a smart assistive device for the elderly

**Table 4:** Statistical inferences of activity level in the elderly.

Instance-ID	Polynomial	Least I999(1)	Most I001(1)	Values I001(1), I002(1),…, [1017 more]
Parameter (data type)	Min	Max	Average	Deviation
Age (real)	61.850	82.850	66.023	8.377
Time interval (real)	2.650	14.040	6.435	3.172
Active thought (real)	0.001	0.208	0.093	0.042
Work (real)	0.292	0.564	0.428	0.063
Ideal (real)	0.152	0.166	0.160	0.005
Sleep (real)	0.131	0.345	0.236	0.059
Stress (real)	0.010	0.312	0.199	0.074

**Table 5:** Activity-level assessment results.

	*P*_1_	*P*_2_	*P*_3_	*P*_4_
*A*_1_	0.811	0.890	0.920	0.874
*A*_2_	0.842	0.902	0.896	0.880
*A*_3_	0.891	0.913	0.939	0.915
*A*_4_	0.862	0.924	0.958	0.9146
*A*_5_	0.852	0.912	0.967	0.9104

**Table 6:** CDL assessment outcomes in the elderly.

	*P*_1_	*P*_2_	*P*_3_	*P*_4_
CDL = 4	0.520	0.532	0.541	0.531
CDL = 6	0.720	0.731	0.742	0.731
CDL = 8	0.981	0.925	0.920	0.942

**Table 7:** Device error prediction results.

Model	Structure	Eta	Epoch	Training error	Testing error
NN0	3-1	0.1	100	—	—
		0.5	100	—	—
NN1	3-5-1	0.1	100	0.33635	0.34335
		0.5	100	0.29615	0.27612
NN2	3-5-5-1	0.1	100	0.03345	0.03526
		0.5	100	0.02215	0.02341
